# Prenatal Exposure to TCDD Triggers Significant Modulation of microRNA Expression Profile in the Thymus That Affects Consequent Gene Expression

**DOI:** 10.1371/journal.pone.0045054

**Published:** 2012-09-14

**Authors:** Narendra P. Singh, Udai P. Singh, Hongbing Guan, Prakash Nagarkatti, Mitzi Nagarkatti

**Affiliations:** Department of Pathology, Microbiology & Immunology, University of South Carolina School of Medicine, Columbia, South Carolina, United States of America; Tulane University, United States of America

## Abstract

**Background:**

MicroRNAs (miRs) are a class of small RNAs that regulate gene expression. There are over 700 miRs encoded in the mouse genome and modulate most of the cellular pathways and functions by controlling gene expression. However, there is not much known about the pathophysiological role of miRs. TCDD (2,3,7,8-tetrachlorodibenzo-*p*-dioxin), an environmental contaminant is well known to induce severe toxicity (acute and chronic) with long-term effects. Also, in utero exposure of fetus to TCDD has been shown to cause thymic atrophy and alterations in T cell differentiation. It is also relevant to understand “the fetal basis of adult disease” hypothesis, which proposes that prenatal exposure to certain forms of nutritional and environmental stress can cause increased susceptibility to clinical disorders later in life. In the current study, therefore, we investigated the effects of prenatal exposure to TCDD on miR profile in fetal thymocytes and searched for their possible role in causing thymic atrophy and alterations in the expression of apoptotic genes.

**Methodology/Principal Findings:**

miR arrays of fetal thymocytes post exposure to TCDD and vehicle were performed. Of the 608 mouse miRs screened, 78 miRs were altered more than 1.5 fold and 28 miRs were changed more than 2 fold in fetal thymocytes post-TCDD exposure when compared to vehicle controls. We validated the expression of several of the miRs using RT-PCR. Furthermore, several of the miRs that were downregulated contained highly complementary sequence to the 3′-UTR region of AhR, CYP1A1, Fas and FasL. Also, the Ingenuity Pathway Analysis software and database was used to analyze the 78 miRs that exhibited significant expression changes and revealed that as many as 15 pathways may be affected.

**Conclusions/Significance:**

These studies revealed that TCDD-mediated alterations in miR expression may be involved in the regulation of its toxicity including cancer, hepatic injury, apoptosis, and cellular development.

## Introduction

MicroRNAs (miRs) regulate gene expression. They are endogenously encoded in the genome and belong to a class of small RNAs. The miRs are initially transcribed as long primary transcripts (pri-miRs), which are subsequently modified into pre-miRs that possess approximately 70 nucleotide stem loop structures and are present within the nucleus. Pre-miRs are then exported from the nucleus to the cytoplasm and are modified again to mature miRs consisting of 19–25 nucleotide duplexes. Mature miRs are then incorporated into the RNA-induced silencing complex (RISC). The mature miRs guide the RISC to bind complementary sequences of the target genes most often in the 3′ UTR region [1]. There are approximately 17,000 miR sequences listed in the miR database [2].

The biological significance of miR generation is evident by their ability to regulate gene expression causing serious effects on various physiological, pathological, and other biological mechanisms and functions. The miRs have been shown to regulate up to 30% of the mammalian genes [3] suggesting that most cellular pathways are potentially regulated by miRs [4], [5], [6]. The effect of miRs can be of various degrees from mild to very strong. The strong effect of miRs is evident from the lethality of knockout mice that lack any of the enzymes responsible for miR production, such as Ago2, Dicer, and Drosha. Some of cellular processes regulated by miRs include apoptosis, cell growth, fat storage, insulin secretion, and cancer initiation and progression [4], [5], [6]. miRs may play a significant role in responses to xenobiotic chemicals and their role in causing various health associated problems and ailments. Fukushima et. al. have shown that exposure of rats to liver toxicants such as acetaminophen or carbon tetrachloride caused alteration in the expression of various miRs [7]. In another report, tamoxifen, a potent hepatocarcinogen, was shown to increase the expression of several miRs associated with oncogenes [8]. There are reports demonstrating that cigarette smoking can cause changes in miR expression profile [9]. It has also been shown that mothers smoking cigarettes can exhibit changes in expression levels of miRs related to growth and developmental processes [10]. Similarly, other chemicals, such as bisphenol A, have been shown to cause alteration in miR expression *in vitro* [11]. There are also reports suggesting that drug-metabolizing enzymes such as CYP family genes are targeted by certain miRs [6], [12]. These reports suggest that miRs may regulate the toxicity mediated by environmental chemicals.

TCDD (Dioxin) belongs to a group of halogenated aromatic hydrocarbons and is well known for its immunotoxic and carcinogenic properties [13], [14], [15], [16], [17], [18], [19], [20]. Recent epidemiological and experimental evidence has led to the advancement of “the fetal basis of adult disease” hypothesis, which suggests that malnutrition or exposure to environmental stress during pregnancy, may have a long lasting impact on the developing fetus, leading to increased susceptibility to a wide range of diseases later in life, including cancer, hypertension, cardiovascular, and autoimmune diseases [21], [22]. We and others have shown that exposure to TCDD during pregnancy severely affects the immune system of the mothers and their fetuses by triggering apoptosis in thymic T cells, altering T cell subsets and functions, as well as expression of co-stimulatory molecules [14], [23]. The majority of biological effects of TCDD leading to immunotoxicity and associated deleterious effects are mediated by aryl hydrocarbon receptor (AhR) [24]. The necessity of AhR for TCDD-induced toxicity was revealed by experiments using AhR-null mice, which exhibited resistance to toxicity [25], [26], [27]. TCDD exposure elicits the upregulation of a large number of genes in an AhR-dependent manner [28] and it is predicted that some of these AhR target genes are directly responsible for the induction of dioxin toxicity.

Given that a large number of genes are regulated by miRs and that most of the biological processes including responses to TCDD are expected to be regulated by miRs, it is reasonable to hypothesize that there are certain types of miRs that regulate TCDD-mediated toxicity. Also, previous studies from our laboratory have suggested that prenatal exposure to TCDD causes marked changes in the immune response [14], [29], [30]. Therefore, we searched for miRs that are dysregulated in fetuses following prenatal exposure to TCDD, which may be involved in the TCDD-induced toxicity. Our studies demonstrate for the first time that prenatal exposure to TCDD caused significant changes in the fetal thymocyte miR expression profile. The dysregulation of miRs in fetuses by TCDD may have long-lasting effects in adult life and contribute towards dysregulation in the immune response.

## Results

### Cluster Analysis of miR Profile in Fetal Thymocytes Post-TCDD Exposure

**Figure 1:**
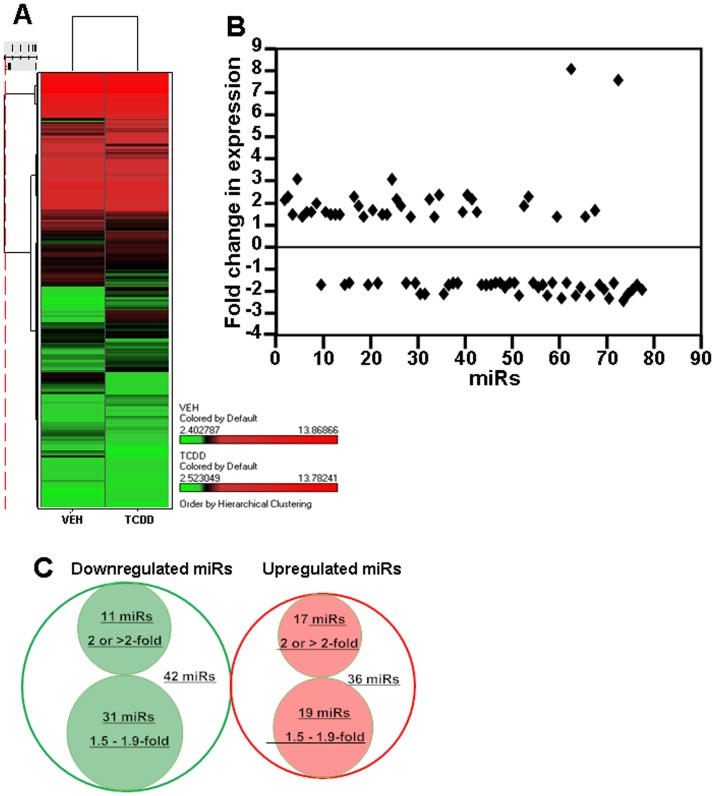
Heat map of miR expression profile in fetal thymi post-TCDD exposure. A. Heat map depicting miR expression profile in fetal thymi exposed to TCDD with that of fetal thymi exposed to vehicle (control). The expression pattern (green to red) represents the spectrum of downregulated to upregulated expression pattern of miRs. B. Depicts fold change expression profile of miRs post-TCDD exposure in comparison to vehicle. Of the ∼251 miRs dysregulated, a significant number of miRs showed more than 1.5 fold change (upregulated or downregulated) in their expression profile. C. Venn diagram illustrating TCDD-mediated downregulated miRs (green circle) and upregulated miRs (red circle) when compared to vehicle.

Raw data obtained from miR arrays of fetal thymocytes post-TCDD or vehicle exposure were analyzed for miR expression. To this end, cluster analysis of 608 miRs was performed using Ward’s method in vehicle- and TCDD-treated samples and data were represented as columns. Similarity measure of miRs of the two groups was done using Half Square Euclidean Distance method and ordering function of miRs was done on the basis of Input rank. The visualization of cluster analysis of miRs have been shown as a dendrogram (a tree graph) based on the similarity between them (Fig 1A) and their expression pattern was reflected in a range from +13.8 to −2.5 (Fig 1A).

### Differential Expression (Fold Change) of miRs

Differential (upregulated or downregulated) expression of miRs was analyzed using 2-sample t-test method. The significance of analysis of microarrays was performed using Kaplan-Meier method. A p-value of <0.01 in the t-test was considered significant. Of the total 608 miRs screened, 251 miRs showed 1 or more than 1 fold upregulated or downregulated expression. There were 78 miRs that showed more than 1.5 fold change and 28 miRs with 2.0 or greater fold differential expression in TCDD group when compared to the vehicle (Fig 1B–C). Expression of miRs, 1.5-fold or higher, was considered positive and this criterion was used for all further analyses. Together, these data demonstrated that TCDD-mediated effects on miR regulation were moderate in terms of fold change although a significant number of miRs showed changes thereby implicating their role in altered gene expression in thymocytes.

### Validation of miR Expression by Real-Time PCR

**Figure 2:**
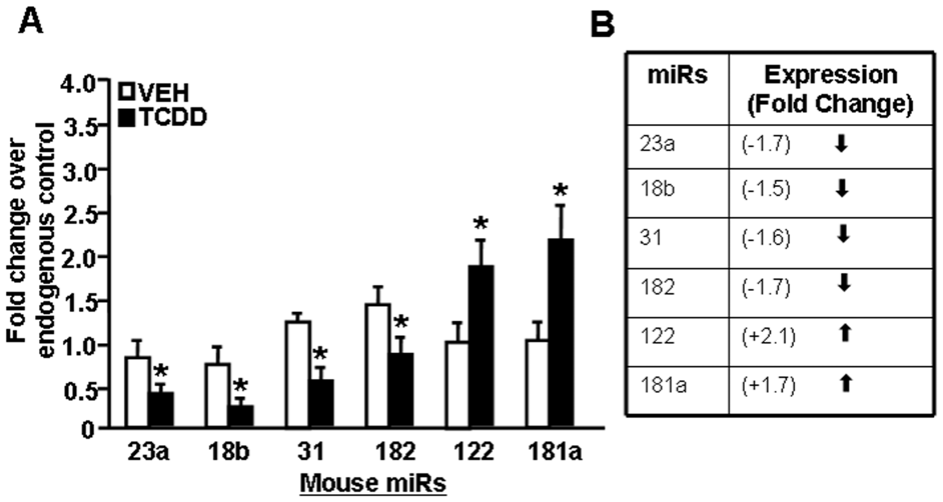
Validation of expression profile of miRs (miR-23a, -18b, -31, -182, -122, and 181a) in fetal thymi post-TCDD exposure. A, Expression profile of the miRs in fetal thymi was determined using miR-specific primers and by performing Real-Time PCR. Data are depicted as mean ± SEM of three independent experiments. Asterisk (*) in panel A indicates statistically significant (p<0.05) difference between groups compared. In panel B, miR expression profile from miR arrays are depicted.

To validate the miR array data, we studied several differentially expressed miRs (upregulated miRs: miR-122 and miR-181a and downregulated miRs: miR-23a, miR-18b, miR-31, and miR-182). To this end, Real-Time PCR was performed on cDNAs converted from total RNAs from thymocytes treated with TCDD or vehicle as described in Materials and Methods. Real-Time PCR analysis demonstrated upregulated expression of miR-122 and miR-181a in thymocytes treated with TCDD when compared to vehicle-treated thymocytes (Fig 2A–B). Similarly, we observed downregulation of miR-23a, miR-18b, miR-31 and miR-182 in TCDD-treated thymocytes when compared to vehicle controls (Fig 2A–B). Thus, the Real-Time PCR data validated the expression profiles obtained from the arrays.

### TCDD-regulated miRs Affect Various Pathways

**Figure 3:**
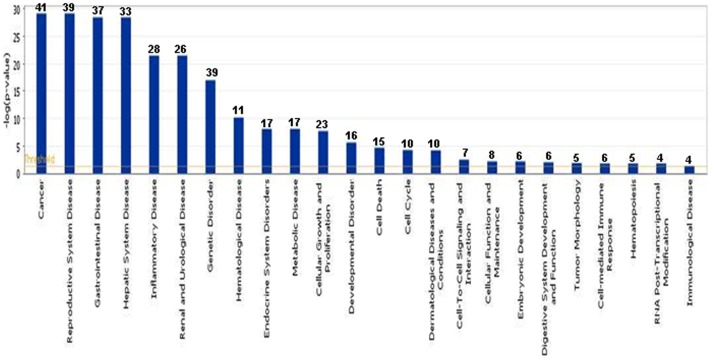
TCDD-regulated miRs and their association with functional networks. TCDD-induced up- or down-regulated (more than 1.5 fold change) miRs were analyzed using IPA software and the database (Ingenuity Systems, Inc). The data presented in the graph demonstrates various pathways regulated by TCDD-induced miRs. On Y-axis, –log(p-value) represents significance of function by random chance (IPA software, Ingenuity Systems, Inc). Number over each bar represents number of miRs involved in pathways.

**Figure 4:**
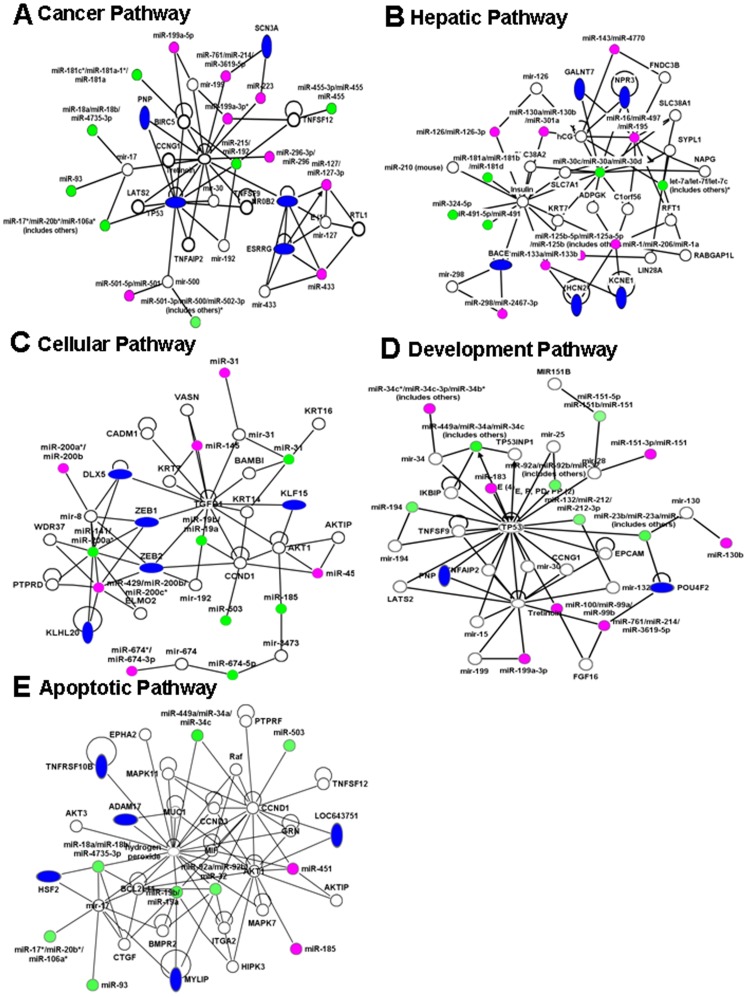
TCDD regulated miRs and their association with various pathways. TCDD-regulated miRs as described in Fig 3 were analyzed using IPA software and the database (Ingenuity Ssytems, Inc). A, miRs involved in cancer pathway, B, hepatic pathway, C, cellular pathway, D, development pathway, and E, apoptotic pathway. In Figure 4, thin line empty circles represents mature miRs with various functions, thick line empty circles represents various genes, magenta circles represent upregulated mature miRs, green circles represent downregulated mature miRs, and blue ovals represent various genes involved in the pathways.

Next, we performed further analysis of miR expression in TCDD-exposed fetal thymi when compared to controls. We selected miRs expressing more than 1.5 fold change for further analysis. To this end, 78 miRs were analyzed using Ingenuity Pathway Analysis (IPA) software and database (Ingenuity Systems, Inc). The analysis revealed that there were as many as 15 pathways that may be affected by various miRs dysregulated by TCDD in fetal thymi (Fig 3). There were 41 miRs involved in cancer-associated pathways, 39 miRs in reproductive system diseases, 37 miRs in gastrointestinal diseases, and 33 in hepatic system diseases (Fig 3). Similarly, as shown in Fig 3, there were several miRs that showed significant alterations involved in other pathways such as inflammatory diseases (28 miRs), renal and urological diseases (26 miRs), genetic disorders (39 miRs), hematological disease (11 miRs), endocrine system diseases (17 miRs), metabolic diseases (17 miRs), cellular growth and proliferation (23 miRs), developmental disorders (16 miRs), cell death (15 miRs), and cell cycle (10 miRs). Upon further analysis of some of the pathways, TCDD-regulated miRs were observed participating in cancer pathway (Fig 4A), hepatic pathway (4B), cellular pathway (Fig 4C), developmental pathway (Fig 4D), and apoptotic pathway (Fig 4E). There were also miRs that were observed to be involved in early signaling, immunotoxic, and apoptotic pathways (Table 1). Together, the analysis revealed that TCDD affects several miRs that play a role in regulating a large number of genes that participate in various pathways influencing early signaling, as well as physiological and metabolic functions, associated with various health-related disorders.

**Table 1:** TCDD-mediated downregulation of miRs and their role in apoptosis and immunotoxicity.

miRs	Fold change	Role in Apoptotic pathways and Immunotoxicity
**miR-27a**	**(>1.5)**	Regulate AhR gene expression
**miR-28**	**(>1.6)**	Regulate AhR gene expression
**miR-29a**	**(>1.5)**	Regulate AhR gene expression
**miR-182**	**(>1.7)**	Regulate AhR gene expression
**miR-203**	**(>1.5)**	Regulate AhR gene expression
**miR-290**	**(>1.5)**	Regulate AhR gene expression
**miR-31**	**(>1.6)**	Regulate CYP1A1 gene expression
**miR-101b**	**(>1.5)**	Regulate CYP1A1 gene expression
**miR-335**	**(>1.5)**	Regulate CYP1A1 gene expression
**miR-23a**	**(>1.7)**	Regulates Fas expression
**miR-23b**	**(>1.9)**	Regulates Fas expression
**mmu-let-7e**	**(>1.9)**	Regulates FasL expression
**miR-18b**	**(>1.5)**	Regulates FasL expression
**miR-98**	**(>1.8)**	Regulates FasL expression
**miR-200a**	**(>1.8)**	Causes apoptosis
**miR-491**	**(>1.8)**	Induces apoptosis targeting BCL-xL

### Analysis of miRs Associated with Apoptosis and their Differential Expression

There are several reports demonstrating that TCDD induces apoptosis in thymocytes leading to thymic atrophy [14], [20], [29], [31], [32], [33], [34], [35], [36], [37], [38]. Therefore, we analyzed miRs that were up- or downregulated and potentially associated with apoptotic pathways. Upon analysis of miRs, we observed that at least six miRs associated with apoptotic pathways, were more than 1.5-fold downregulated in fetal thymocytes post-TCDD exposure, when compared to vehicle (Table 1). For example, miR-23a and miR-23b were downregulated in TCDD-treated thymocytes when compared to vehicle-treated thymocytes (Table 1). These miRs have highly complemantary sequence for 3′-UTR region of Fas gene (Table 2) and thus may be involved in Fas regulation. Similarly, we observed downregulated (>1.5-fold) expression of mmu-let (mmu-let-7b, 7c and 7e), miR-18b and miR-98 in fetal thymocytes post TCDD exposure and these miRs possess highly complemantary sequence with FasL 3′-UTR (Table 2) demonstrating that these miRs may be involved in FasL expression. We also observed downregulated (>1.5-fold) expression of two other miRs: miR-200a and miR-491 in TCDD-exposed fetal thymocytes. miR-200a has been reported to play a crucial role in apoptosis [39], whereas miR-491 has been shown to influence apoptosis by targeting BCL-xL gene in colorectal cancer cells [40]. Together, the data obtained from miR analysis showed that TCDD-induced apoptosis may result from dysregulation of miRs associated with apoptotic pathways.

**Table 2:** Showing miRs containing highly complementary sequence for 3′ UTRs of various target genes.

miR-23a and Fas 3′UTR binding3' ccUUUAGGGACCG-UUACACUa 5' mmu-miR-23a||||:: | : |||||||244:5' ugAAAUUUGUAUUAAAUGUGAa 3' Fas
miR-18b and FasL 3′UTR binding3' gauugUCGUGA–UCUAC-GUGGAAu 5' mmu-miR-18b| ||:| ||| | ||||||310:5' uuuuaACCAUUGAAGAAGACACCUUu 3' FasL
miR-31 and CYP1A1 3′UTR binding3' gucgauacgguCGUAGAACGGa 5' mmu-miR-31|| |||||||359:5' ggcacagagguGC-UCUUGCCa 3' CYP1A1
Mmu-let-7e and FasL 3′UTR binding3' uugauauGUUGGAGGAUGGAGu 5' mmu-let-7e| ||:| |||||||350:5' gguggguCUACUUACUACCUCa 3' FasL

### Expression of AhR, CYP1A1, Fas, and FasL in Fetal Thymocytes

**Figure 5:**
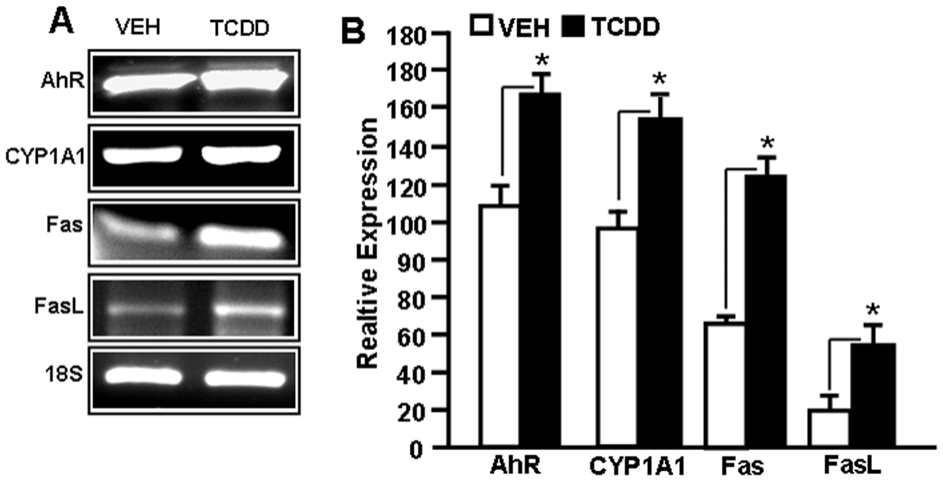
Expression of AhR, CYP1A1, Fas, and FasL in fetal thymocytes post-TCDD exposure. A, Fetal thymi exposed to TCDD as described in Fig 1 were analyzed for the expression of AhR, CYP1A1, Fas, and FasL using RT-PCR. In panel B, RT-PCR data are presented as percentage of 18S expression with the latter being considered as 100%. Data are depicted as mean ± SEM of three independent experiments. Asterisk (*) in panel B indicates statistically significant (p<0.05) difference between groups compared.

To further corroborate that in the same samples of fetal thymi that were analyzed for miRs, we could see changes in the expression of AhR, CYP1A1, Fas, and FasL genes as reported earlier [20], [29], [32], [33], [38], RT-PCR analysis was performed. Data obtained from RT-PCR showed upregulated expression of AhR, CYP1A1, Fas, and FasL in TCDD-treated thymocytes (Fig. 5A–B). We observed similar 18S (a house keeping gene) expression between TCDD- and vehicle-treated thymocytes (Fig. 5A–B). These data correlated with the expression of various miRs that regulate the expression of such genes. For example, downregulation of miR-182 that has highly complementary sequence with AhR 3′-UTR was responsible for increased expression of this target gene. Similarly, the expression of miRs, miR-31, miR-23a, and miR-18b which regulate CYP1A1, Fas, and FasL respectively, were decreased following TCDD treatment.

### Analysis of miRs Associated with Fas and FasL Expression

Previous studies from our laboratory and others have reported TCDD- mediated upregulation in the expression of Fas and FasL in activated T cells and thymic cells [13], [14], [20], [29], [32], [38]. We also reported that Fas/FasL-mediated apoptosis may be one of the important mechanisms causing thymic atrophy and apoptosis in T cells [14], [20], [29], [31], [32], [33], [34], [35], [36], [37], [38]. In this context, we analyzed miRs (miR-23a and mmu-let-7e) that were downregulated and are associated with Fas and FasL expression respectively. Upon analysis of highly complementary sequence of miR-23a and mmu-let-7e using microRNA.org and/or TargetScanMouse 5.1databases, highly complementary sequence of miR-23a with 3′-UTR region of Fas and highly complementary sequence of mmu-let-7e with FasL gene was observed (Table 2). The data obtained from miR analysis and highly complementary sequence property of miR-23a and mmu-let-7e demonstrated that TCDD may regulate Fas/FasL expression via downregulating miRs (miR-23a and mmu-let-7e).

### Analysis of mmu-let-7e and FasL Expression

**Figure 6:**
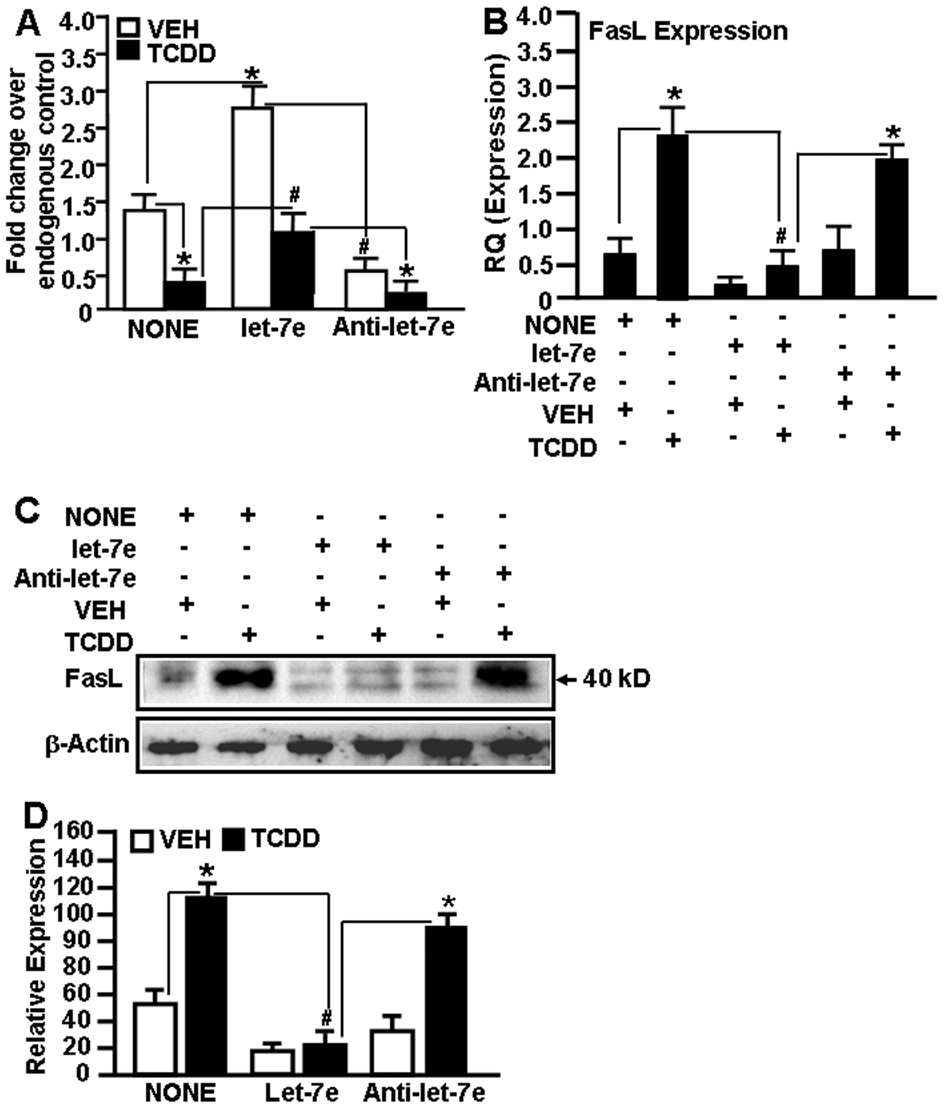
Expression of FasL in EL4 cells in the presence or absence of mmu-let-7e and post-vehicle or TCDD treatment. A, EL4 cells, not transfected or transfected with mature mmu-let-7e and exposed to vehicle or TCDD, were analyzed for the expression of FasL by performing Real-Time PCR. In panel B, FasL expression was determined by performing Real-Time PCR on cDNAs generated from EL4 cells not transfected or transfected with mmu-let-7e or anti-mmu-let-7e or negative control (-Ve) for mmu-let-7e and exposed to vehicle or TCDD. Real-Time PCR data are presented as fold change in expression. Data are depicted as mean ± SEM of at least three independent experiments. Asterisks (* and #) in panel A and B indicate statistically significant (p<0.05) difference between groups compared. Panel C, FasL expression at the protein level in EL4 cells not transfected or transfected with mature mmu-let-7e or anti-mmu-let-7e and treated with vehicle or TCDD. Data are depicted as mean ± SEM of at least three independent experiments in panel D. Asterisks (* and #) in panel D indicate statistically significant (p<0.05) difference between groups compared.

To further understand the role of mmu-let-7e in FasL expression, we performed a series of *in vitro* assays. To this end, EL4 T cells, not transfected or 48 hrs post-transfection with mature mmu-let-7e or anti-mmu-let-7e, were cultured in the absence or presence of TCDD for 24 hrs. The expression of mmu-let-7e was determined in vehicle- or TCDD-treated cells by performing Real-Time PCR and expression of FasL was determined by performing Real-Time PCR and Western blotting. EL4 cells not transfected (NONE) but treated with TCDD showed significantly downregulated expression of mmu-let-7e, when compared to vehicle-treated EL4 cells (Fig 6A). However, there was significantly higher expression of mmu-let-7e in EL4 cells that were transfected with mmu-let-7e and treated with vehicle or TCDD, while transfection with anti-mmu-let-7e led to down regulation of mmu-let-7e (Fig 6A).

Upon examination of FasL expression in these various forms of treatment in EL4 cells using Real-Time PCR, significantly upregulated expression of FasL was observed in TCDD-treated non-transfected EL4 cells, when compared to vehicle-treated non-transfected EL4 cells (Fig 6B). Upon transfection of EL4 cells with mmu-let-7e and treatment with TCDD, there was significant downregulation of FasL expression when compared to non-transfected EL-4 cells treated with TCDD (Fig 6B). In contrast, EL4 cells transfected with anti-mmu-let7e and treated with TCDD showed marked upreglulation in the expression of FasL when compared to EL4 cells transfected with mmu-let-7e and treated with TCDD (Fig 6B). Furthermore, upon examination of FasL expression in these treated cells at the protein-level, we obtained similar results (Fig 6C–D). These data demonstrated that TCDD-mediated downregulation of mmu-let-7e expression may contribute towards upregulated expression of FasL and thus mmu-let-7e may regulate the expression of FasL.

### TCDD-induced Downregulation of mmu-let-7e Affects FasL Expression

**Figure 7:**
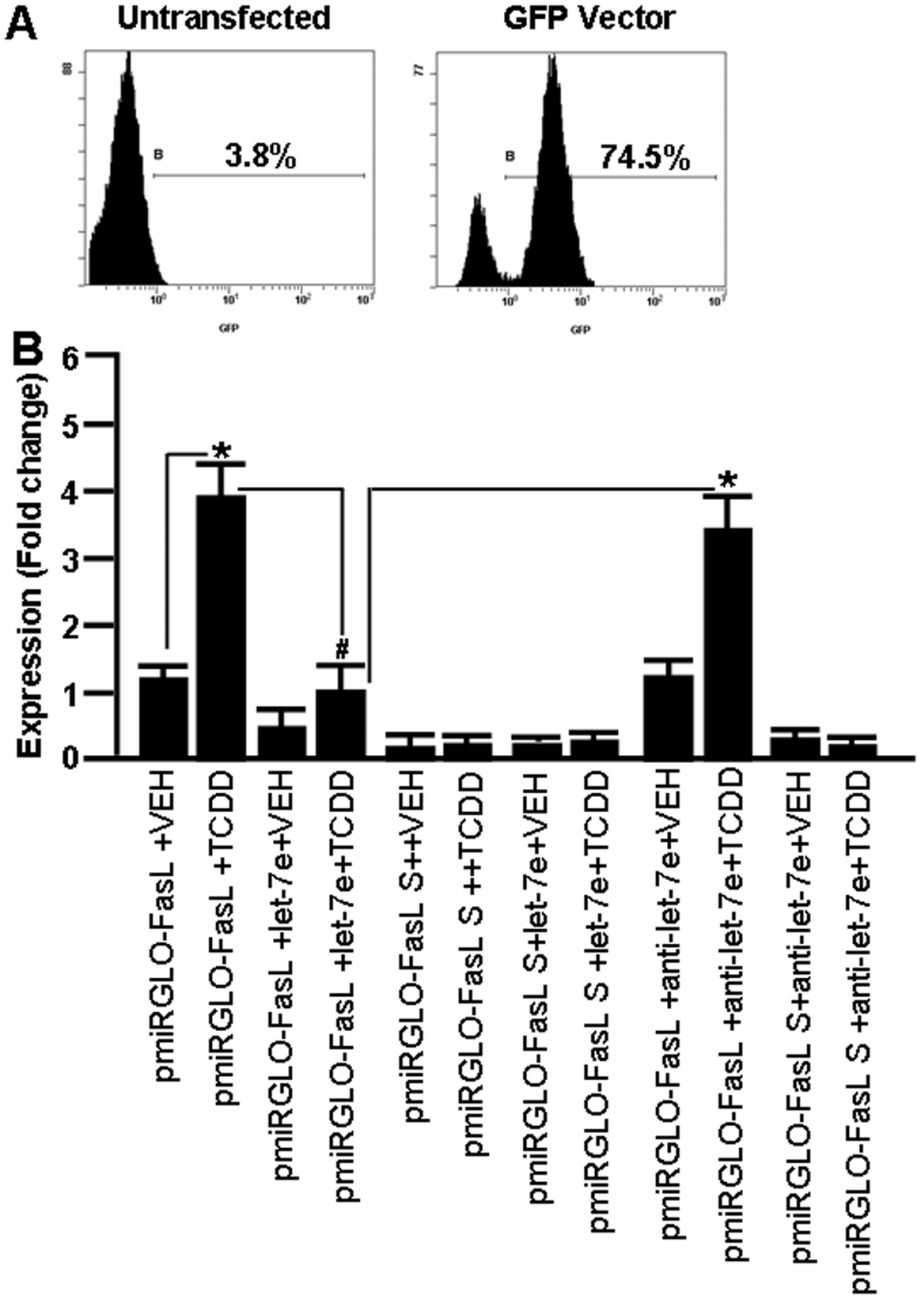
Expression of luciferase in EL4 cells in the absence or presence of FasL UTR containing mmu-let-7e binding site post-vehicle or TCDD treatment. A: Determination of transfection efficiency of EL4 cells. EL4 cells were co-transfected with GFP containing vector and pmiRGLO-FasL plasmid and were analyzed using flow cytometry. There was more than 74% transfection of EL4 cells. B: Real-Time PCR was performed to determine luciferase expression by performing luciferase assays of EL4 cells not transfected or transfected with pmiRGLO-FasL or pmiRGLO-FasL S and exposed to vehicle or TCDD. Luciferase expression data are presented as fold change in expression. Data are depicted as mean ± SEM of three independent experiments. Asterisks (* and #) in panel B indicate statistically significant (p<0.05) difference between groups compared.

To understand TCDD-regulated expression of mmu-let-7e and its role in regulation of FasL expression, FasL UTR region containing normal mmu-let-7e complementary region or scramble FasL UTR region were cloned into pmiRGLO luciferase expression vector and the clones were designated as pmirGLO-FasL and pmirGLO-FasL-S respectively (as described in Materials and Methods). EL4 cells not transfetcted or transfected with pmirGLO-FasL or pmirGLO-FasL-S plasmids or transfected with mature mmu-let-7e or anti-mmu-let-7e were treated with vehicle or TCDD (100 nM/ml) for 24 hrs. There was ∼75% transfection of EL4 cells (Fig 7A). Upon analysis of luciferase expression, the main summary findings were as follows: there was significantly upregulated expression of luciferase in EL4 cells transfected with pmirGLO-FasL in the presence of TCDD, when compared to EL4 cells treated with vehicle (Fig 7B). In contrast, there was significant downregulation in the expression of luciferase in EL4 cells transfected with pmiR_GLO-FasL and mmu-let-7e following TCDD treatment (Fig 7B), whereas, in EL4-cells transfected with pmiR-GLO-FasL and anti-mmu-let-7e, there was significant increase in FasL expression in the presence of TCDD (Fig 7B).

### Differential Expression of miRs Associated with AhR and CYP1A1 Gene and Immunotoxicity

AhR signaling has been shown to be an important player in TCDD-induced thymic atrophy and immunotoxicity. Also, CYP1A1 induction is a hallmark of AhR activation by TCDD [18], [41], [42], [43], [44]. To understand the role of TCDD-induced miRs in regulation of AhR and CYP1A1 expression, we sought to identify miRs that were up- or downregulated by TCDD in fetal thymocytes. Upon miR analysis, we observed several miRs that were downregulated (>1.5-fold) in thymocytes post-TCDD exposure thereby indicating that these miRs may be associated with regulation of AhR and CYP1A1 vis-a-vis immunotoxicity (Table 1). There were 6 downregulated (>1.5-fold) miRs (miR-27a, -28, -29a, -182, -203, and -290) in TCDD-treated thymocytes (Table 1) and these miRs showed highly complementary sequence with 3′-UTR of AhR gene indicating that these miRs may be involved in AhR expression in thymocytes. There were three other miRs (miR-31, -101b, and -335) that were also downregulated (>1.5-fold) in TCDD-treated thymocytes (Table 1) and showed highly complementary sequence with 3′-UTR of CYP1A1 gene. These data demonstrate that TCDD-mediated downregulation of miRs in fetal thymocytes may play a role in the induction of AhR and CYP1A1 in thymocytes post-TCDD exposure.

### Differential Expression of miRs Associated with Cancer

TCDD has previously been shown to cause cancer in various species [45], [46], [47], [48], [49]. There are also reports demonstrating the role of miRs in generation, development, and progression of various types of cancer [39], [50], [51], [52], [53], [54], [55], [56], [57]. To this end, we analyzed the expression of certain miRs known to play a role in the regulation of cancer. There were about 25 cancer-associated miRs that showed dysregulated expression pattern in fetal thymocytes post-TCDD exposure. Of these, there were 11 miRs that were more than 1.5-fold upregulated and 14 miRs that were downregulated in thymocytes exposed to TCDD (Table 3). Upon further analysis, based on previous reports, we observed that these dysregulated miRs directly/indirectly may be involved in generation, development, and progress of various types of cancers (Table 3). The details of miRs, their expression, and their role in development of various cancers are as described in Table 3.

**Table 3:** TCDD-mediated upregulation or downregulation of miRs and their role in cancer.

miRs	Fold changeUpregulated	Role in Cancer
**miR-122**	**(>1.7)**	Role in liver metabolism and cancer
**miR-125a**	**(>1.7)**	Represses Mesenchymal Morphology in Ovarian Cancer cells
**miR-127**	**(>2.4)**	Role in cancer development
**miR-145**	**(>3.2)**	C-Myc expression through p53
**miR-199a**	**(>3.1)**	Regulates MET Protooncogene and effects NF-KB expression
**miR-379**	**(>1.9)**	Affects Brain Neuronal development
**miR-451**	**(>3.2)**	Erythroid differentiation
**miR-126**	**(>2.5)**	Angiogenic signaling and controls blood vessel development
**miR-143**	**(>1.9)**	Regulates ERK5 signaling and smooth muscle cells
**miR-298**	**(>1.8)**	Regulates CYPA3 expression
**miR-486**	**(>7.7)**	Regulates Kinase activity and tumor progression
	**Downregulated**	
**miR-31**	**(>1.6)**	Promotes lung cancer
**miR-34a**	**(>1.9)**	Inhibits prostate cancer and metastasis by repressing CD44
**miR-181c**	**(>1.6)**	Epigenetically silenced miRNA and involved in gastric cancer
**miR-700**	**(>2.9)**	Cancer development
**miR-671**	**(>2.1)**	Cancer growth and development
**miR-669**	**(>1.9)**	C-Myc expression through p53
**miR-500**	**(>2.2)**	Regulates MET Protooncogenes and effects NF-KB
**miR-491**	**(>1.8)**	TGF-Beta inducer
**miR-466**	**(>1.8)**	Mammary tumor development
**miR-466c**	**(>1.7)**	Tumor growth
**miR-449a**	**(>2.1)**	Breast cancer development and inhibits cell proliferation
**miR-134a**	**(>1.8)**	Cancer development
**let-7b**	**(>1.5)**	Regulates RAS expression
**let-7c**	**(>1.7)**	Regulates neural stem cell proliferation
**let-7e**	**(>1.9)**	Role in Colorectal and other types of cancer

## Discussion

TCDD toxicity has been well characterized to be regulated by signaling through the AhR leading to the induction of a wide range of genes that express DREs on their promoters [20], [26], [44], [58], [59], [60], [61], [62]. However, it is not clear whether the toxic effects of TCDD may also be regulated by certain miRs. The possibility that miRs might modulate mRNA levels and subsequent toxicity by TCDD has not been fully explored. Recently, a few studies have begun exploring such mechanisms [63], [64]. One study reported that miR-27b related to AhR-regulated genes increased CYP1B1 levels [6]. In another study, it was noted that treatment with TCDD *in vivo* caused few changes in miR levels in mouse or rat livers, and those changes that were statistically significant were of modest magnitude [63]. These data are consistent with our studies where we noted that the magnitude of change in miR expression following TCDD treatment in most instances was 1.5 to 2 fold and only a few miRs showed 3–8 fold change. The fact that liver may be more refractory was also indicated in another study in which it was noted that AhR activation by benzo(a)pyrene (BaP) did not cause significant changes in miRs of the liver but altered the miR profiles in the lung [65]. The miRs that were altered by BaP were involved in immune response, cell proliferation and cell cycle [65]. Thus, it is likely that the AhR-agonist mediated changes in miRs may be organ-specific.

While, the immunotoxic effects of prenatal exposure to TCDD on fetal thymocytes have been well characterized, there are no reports on such effects of TCDD on miR profiles. Understanding the role of various miRs in neonatal mice post-TCDD exposure may shed light on the “fetal basis of adult disease” hypothesis. This hypothesis proposes that many chronic diseases including autoimmune diseases during adult stage of life may be the result of prenatal exposure to nutritional, environmental or other forms of stress [21], [22], [66]. In this study, therefore, we sought to examine miR profile in fetuses post-TCDD exposure.

The cluster analysis data of miRs showed that TCDD caused significant changes in miR expression profile in fetal thymi when compared to vehicle-treated thymi. Of the miRs screened, 78 miRs were altered more than 1.5 fold and 28 miRs were altered two fold or more, post-TCDD exposure. We further validated the expression profile of some select miRs by performing Real-Time PCR. All the miRs that we analyzed by Real-Time PCR corroborated the data obtained from miR array analysis. Furthermore, the relationship of miRs and their target gene expression was also verified. For example, miRs that showed highly complementary sequence with 3′UTR of AhR, CYP1A1, Fas, and FasL genes were downregulated by TCDD in fetal thymi and the data obtained from RT-PCR showed upregulated expression of the above genes in fetal thymi post-TCDD exposure.

TCDD is known to induce toxicity in a wide range of tissues or organs. In this study, we noted TCDD-induced upregulation of the following miRs (miR-122, -125a, -151, 181a, -200b, -206, -322, -345, -367b, -296, and -466i) and their expression profile varied from 1.5 to 2.0 fold. As shown in Table 4, these upregulated miRs control expression of various genes in different tissues including thymus, liver, mesenchyma, skeletal muscle, endothelial cells, heart muscle, etc. and affect various physiological and biological mechanisms. For example, miR-122 that showed increased expression (>2.1 fold) in fetal thymi post-TCDD exposure, plays an important role in liver metabolism, toxicity, and cancer [64], [67]. Furthermore, there were also about 7 miRs that were very distinct in their downregulated (varied from 1.5 to 2.1 fold) expression profile post-TCDD exposure. These miRs were miR-15a, -19a, -34a, -140, -146b, -192, and -449a and are expressed in various tissues such as breast, cartilage, endothelial cells, embryonic tissues, etc. These downregulated miRs have been shown to control genes that are involved in various physiological functions in these tissues [68], [69], [70], [71], [72], [73], [74], [75], [76], [77].

**Table 4:** TCDD-mediated miRs expressed in various tissues and their role.

miRs	Fold changeUpregulated	Role in various Tissues
**miR-122**	**(>1.7)**	Role in liver metabolism
**miR-125a**	**(>1.7)**	Represses mesenchymal morphology in ovarian cancer cells
**miR-151**	**(>2.1)**	Involved in osteoclastogenesis and etiology of osteoporosis
**miR-181a**	**(>1.7)**	Modulator of T cell sensitivity and selection
**miR-200b**	**(>1.9)**	Epithelial to mesenchymal transition (EMT) cancer tissues
**miR-206**	**(>1.6)**	Regulates connexin43 during skeletal muscle development
**miR-322**	**(>2.0)**	Muscle differentiation and promotes cell cycle quiescence
**miR-345**	**(>1.5)**	Post-transcriptional regulation of gene in multicellular organisms
**miR-367b**	**(>2.0)**	Regulates thrombospondin-2 (Thbs-2) in placenta
**miR-466i**	**(>1.9)**	Regulates heart development
**miR-296**	**(>1.9)**	Regulates growth factor receptor overexpression in angiogenic endothelial cells
	**Downregulated**	
**miR-15a**	**(>1.9)**	Promotes endocrine resistance to breast cancer downregulating Bcl-2
**miR-19a**	**(>1.7)**	Mediates the suppressive effect of laminar flow on cyclin D1 expression in human umbilical vein endothelial cells
**miR-140**	**(>1.9)**	Expressed in cartilage tissues of mouse embryos and targets histone deacetylase-4 gene
**miR-146b**	**(>1.6)**	Inhibits glioma cell migration and invasion by targeting MMPs
**miR-192**	**(>1.9)**	Involved in the p53 tumor suppressor network with significant effect on cell cycle control and cell proliferation
**miR-449a**	**(>2.1)**	Targets HDAC-1 and induces growth arrest in prostate cancer

As miRs function as negative regulators of gene expression, we considered miRs that were altered more than 1.5 fold, as also reported in other studies [63]. The downregulated expression of the following miRs (miR-27a, -28, -29, -182, -203, and 290) in fetal thymi post- TCDD exposure indicated that these miRs may regulate expression of genes modulated by TCDD. Upon analysis, we observed that these miRs possessed highly complementary sequence with 3′UTR region of AhR gene. TCDD initiates its early signaling via interaction with AhR present in cytosol [20], [26], [28], [38], [61], [78], [79], [80], [81]. TCDD has also been shown to upregulate AhR expression [20], [26], [28], [38], [61], [78], [79], [80], [81]. Moreover, TCDD-AhR interactions participate in regulation of various genes [20], [26], [28], [38], [61], [78], [79], [80], [81]. Thus, downregulated expression of these miRs (miR-27a, -28, -29, -182, -203, and 290) post-TCDD exposure suggests that they may be involved in further inducing the AhR in fetal thymi. We also observed upregulated expression of AhR in fetal thymi post-TCDD exposure. Similarly, we also observed downregulated expression of some other miRs (miR-31, -101b, and -335) in fetal thymi post-TCDD exposure. Using microRNA.org database for prediction of miR targets, we observed that these miRs possessed highly complementary sequence for 3′UTR of CYP1A1 gene, which may explain, at least in part, the ability of TCDD to induce CYP1A1 gene. Also, CYP1A1 plays a significant role in metabolic processes and toxicity caused by TCDD, inasmuch as, mice deficient in CYP1A1 are resistant to high-dose TCDD-induced lethality [20], [26], [28], [38], [61], [78], [79], [80], [81], [82]. Together, these data demonstrated that a large number of miRs that are downregulated in fetal thymi by TCDD may control the expression of genes involved in toxicity.

There were at least six miRs (miR-23a, -23b, -18b, -98, 200a, and -491) that were significantly downregulated (Table 1) in fetal thymi when compared to vehicle. miR-23a and miR-23b possessed highly omplementary sequence with Fas 3′UTR region (Table 2) whereas, miR-18b and miR-98 showed highly complementary sequence with FasL 3′UTR region (Table 2). We also observed significant downregulation of several mmu-let-7 (mmu-let-7b, let-7c, and let-7e) miRs that possess highly complementary sequence with FasL 3′UTR (Table 2). Previous studies from our laboratory have demonstrated that TCDD-induced thymic atrophy in the adult and fetus may result, at least in part, from induction of apoptosis [20], [29], [33], [37]. We have also reported that such apoptosis may be induced through the extrinsic pathway by the induction of Fas and FasL in thymocytes [20], [38]. Also miR-200a has been shown to regulate apoptosis [83], whereas miR-491 has been shown to induce apoptosis by targeting Bcl-xL gene [40]. Thus, these miRs may directly/indirectly be involved in apoptosis of thymic cells leading to thymic atrophy.

TCDD has also been shown to cause cancer in various species and it is also considered to be a potential carcinogen in humans [49]. There are reports demonstrating that TCDD exposure of mice triggers cutaneous papillomas and squamous cell carcinoma [49]. In another report, prenatal TCDD exposure of rats was shown to make them susceptible to breast cancer [46]. TCDD has also been shown to promote liver cancer [84]. miRs have been shown to influence signaling pathways leading to development of various types of cancer [39], [50], [51], [52], [53], [54], [55], [56], [57]. The data obtained from miR analysis of thymocytes showed several upregulated (11 miRs, >1.5 fold) and downregulated (14 miRs, >1.5 fold) miRs that were found be involved in the induction of various types of cancer either directly or indirectly influencing other pathways (Fig 4A and Table 3). For example, miR-127 has been shown to participate in cancer development [85], miR-145 has been shown to control c-Myc expression through p53 [86], miR-199a regulates MET protooncogene and affects NF-KB expression [54], miR-379 affects brain neuronal development [87], [88], miR-451 affects erythroid differentiation [89], miR-126 affects angiogenic signaling and controls blood vessel development [90], miR-143 regulates ERK5 signaling and targets KRAS gene [91], miR-298 regulates CYPA3 expression [92] and miR-486 regulates kinase activity and tumor progression [93].

Similarly, there were at least 14 miRs (miR-31, -34a, -181c, -671, -700, -669, -500, -491, -466, -466c, -449a, -134a, mmu-let-7b, mmu-let-7c, and mmu-let-7e) that were downregulated (>1.5 fold) by TCDD in fetal thymi and these miRs have also been shown directly/indirectly to be associated with various types of cancer development. For example, miR-31 has been shown to promote lung cancer [55]. Also, miR-671 and miR-700 are involved cancer growth and development [94]. miR-669 is involved in c-Myc expression through p53 [95], miR-500 regulates MET protooncogenes and affects NF-kB [96], miR-466 is involved in mammary tumor development, miR-466c is involved in tumor growth [95], miR-449a regulates breast cancer development and inhibits cell proliferation [71], [97], [98] and miR-Let7b plays a role in myeloid leukemia [99]. Together, such data suggested that TCDD affects a large number of miRs that may be directly or indirectly involved in tumor induction and promotion. The precise role of such miRs in TCDD-induced tumorigenesis and toxicity in vivo can be better addressed by using mice deficient in such miRs.

In summary, we demonstrate for the first time that exposure to environmental toxicants such as TCDD during pregnancy can have a significant effect on the miR profile of fetal thymus and thereby influence the regulation of a large number of genes that may affect the development of the immune system. Identification of miRs as targets for TCDD-induced modulation of gene expression offers insights into novel pathways to further understand the mechanisms of toxicity.

## Materials and Methods

### Mice

Pregnant C57BL/6 mice (timed pregnant: vaginal plug day 0) were purchased from Jackson Laboratory.

### Ethics Statement

The mice were cared and maintained in microisolator cages under conventional housing conditions at the AAALAC-accredited University of South Carolina School of Medicine Animal Resource Facility. IACUC committee of University of South Carolina approved the use of mice for this study (IACUC No: 2033 and date of approval: 09-15-11). Six mice were used in each experimental group and the study was repeated three times.

### Chemicals

TCDD was kindly provided by Dr. Steve Safe (Institute of Biosciences & Technology, Texas A&M Health Sciences Center, College Station, Texas). TCDD suspended in corn oil was used in *in vivo* studies. The following reagents including Epicentre’s PCR premix F and Platinum *Taq* Polymerase kits were purchased from Invitrogen Life Technologies (Carlsbad, CA). miRNeasy kit, miScript cDNA synthesis kit, miScript primer assays kit, and miScript SYBR Green PCR kit were purchased from Qiagen (Valencia, CA).

### In Vivo TCDD Exposure

To determine the prenatal effect of TCDD on miR profile in thymic cell populations of fetuses, a single dose of TCDD (10 µg/kg) or vehicle was administered (ip) into pregnant C57BL/6 mice on GD 14, as described previously [14], [29], [30]. On day 3 post TCDD injection, mice were sacrificed, fetal thymi were harvested, and single cell suspensions were prepared. For each treatment group, at least five pregnant mice were used. Because of the small size of the fetal thymus, we combined the thymi from each treatment group to generate a pool of 25–30 pups.

### Preparation of Thymocytes

Thymi from fetuses of TCDD or vehicle-treated groups of mice were harvested and transferred in complete RPMI-1640 medium. Single cell suspensions of thymi were prepared as described earlier [14], [29]. Thymic cell number and viability was determined using a hemocytometer after staining the cells with trypan blue dye and using an inverted phase contrast microscope.

### Isolation of Total miRs and High-throughput miR Arrays

Total RNA including miRs from fetal thymi exposed to TCDD or vehicle was isolated using miRNeasy kit and according to the manufacturer’s instructions (Qiagen, Valencia, CA). The RNA was hybridized on Affymetrix GeneChip (2.0) high-throughput miR arrays. The data generated from miR arrays were analyzed using hierarchical clustering and pathway network analysis for the induction or repression. The expression of miRs was analyzed using 2-sample t-test. A p-value of <0.01 in the t-test was considered significant. A fold-change (FC) of more than 1.5 between vehicle and control samples was considered positive, as reported in other studies [63].

### Real-Time PCR to Validate the Expression of miRs in Thymocytes

To validate the expression of some of the miRs obtained from high-throughput miR array data, we selected 2 upregulated miRs (miR-122 and miR-181b) and 3 downregulated miRs (miR-23a, miR-98, and miR-31). Real-Time PCR assays were performed on cDNA generated from total RNA including miRs isolated from fetal thymocytes exposed to TCDD or vehicle as described earlier. We used miScript primer assays kit (details in Table 5) and miScript SYBR Green PCR kit from Qiagen and followed the protocol of the company (Qiagen, Valencia, CA).

**Table 5:** Real-Time PCR to measure expression level of miRs.

miRBase ID	Target Sequences	Qiagen Cat No
Mmu-miR-23a_st	AUCACAUUGCCAGGGAUUUCC	MS00007266
Mmu-miR-18b_st	UAAGGUGCAUCUAGUGCUGUUAG	MS00011326
Mmu-let-7e_st	UUGAUAUGUUGGAGGAUGGAGU	PM12855 Applied Biosys
Mmu-miR-31_st	AGGCAAGAUGCUGGCAUAGCUG	MS00001407
Mmu-miR-182_st	UUUGGCAAUGGUAGAACUCACACCG	MS00011291
Mmu-miR-122_st	UGGAGUGUGACAAUGGUGUUUG	MS00001526
Mmu-miR-181a_st	AACAUUCAACGCUGUCGGUGAGU	MS00011263

We used StepOnePlus Real-Time PCR system V2.1 (Applied Biosystems, Carlsbad, CA) and at the following conditions: 40 cycles using the following conditions: 15 min at 95°C (initial activation step), 15 s at 94°C (denaturing temperature), 30 s at 55°C (annealing temperature), and 30 s at 70°C (extension temperature and fluorescence data collection) were used. Normalized expression (NE) of miRs was calculated using NE ¼ 2_DDCt, where Ct is the threshold cycle to detect fluorescence. The data were normalized to various miRs against internal control miR and fold change of miRs were calculated against control miR, and treatment group (TCDD) was compared with vehicle group. To define significant differences in miR levels in the thymi of TCDD- or vehicle-treated groups, ANOVA was performed using GraphPad version 4.0 (GraphPad Software, Inc., San Diego, CA). Differences between treatment groups were considered significant when: p<0.05.

### Analysis of miRs and their Association with Various Pathways

For the generation of heatmap and analysis of miR expression, we selected miRs that were up- or downregulated more than 1.5 fold in fetal thymi exposed to TCDD, when compared to vehicle controls. Next, the selected miRs were analyzed for their role in expression of various genes and pathways using IPA software and database (version 15, Ingenuity Systems Inc., CA).

### miR-mRNA Target Interactions

We identified miR-specific mRNA targets using micro RNA.org, TargetScan mouse 5.1, and miRGEN (version 3) software and databases. Computational algorithms supported this task by examining base-pairing rules between miR and mRNA target sites, location of binding sites within the target’s 3′-UTR, and conservation of target binding sequences within related genomes. The details of some of miRs and 3′UTR of their target gene (mRNA targets) are described in Table 2.

### Transfection with Mature mmu-let-7e and Determination of FasL Expression in the Absence or Presence of TCDD

To understand the role of mmu-let-7e in regulation of FasL expression, EL4 cells (5×10^6^) were transfected using Lipofectamine RNAMAX transfection kit from Invitrogen and following Reverse Transfection protocol of the company (Invitrogen). Forty eight hrs post transfection, EL4 cells were treated with vehicle or TCDD (100 nM/ml) for 24 hrs. The expression of FasL was determined in the absence or presence of TCDD. In brief, total RNA from EL4 cells not transfected or transfected with mmu-let-7e or anti-let-7e and treated with vehicle or TCDD were isolated using RNeasy mini kit and following the protocol of the company (Qiagen, Valencia, CA). First strand cDNA synthesis was performed on total RNA (1 µg) and using iScript Kit and following the protocol of the company (Bio-Rad). Real-Time PCR was performed to determine the expression of FasL using mouse FasL-specific sets of primers as described elsewhere [20], [38]. Mouse 18S primer pairs were used as internal control [100].

We also performed Western blotting to determine FasL expression at the protein level in EL4 cells not transfected or transfected with mmu-let-7e or anti-mmu-let-7e and treated with vehicle or TCDD. To this end, we used FasL-specific polyclonal antibody that cross reacts with mouse FasL (Millipore, Temecula, CA). Western blotting was performed following the protocol of the company and as described earlier [20], [100].

### Generation of Reporter Constructs Containing Mouse mmu-let-7e-specific Mouse FasL UTR Region

Reporter construct was generated containing mouse FasL UTR DNA sequences. To this end, we used pmirGLO reporter vector from Promega (Promega Corporation, Madison, WI). pmirGLO reporter vector contains two luciferase genes, 1) firefly luciferase reporter gene (luc2) that generates luminescence in the absence of microRNA and 2) Renilla luciferase reporter gene (hRluc-neo fusion protein coding region) that generates luminescence in presence of microRNA. Mouse FasL-specific UTR region complementary to let-7e was cloned into pmirGLO vector and these were designated as pmirGLO-FasL or pmirGLO-FasL scramble (pmiRGLO-FasL S). The details of the FasL sequences cloned into pmiRGLO are as described below.

#### Oligonucleotides

Both nucleotides of normal and scramble mmu-let-7e-specific FasL UTR regions contain Pmel and Xba1 restriction sites.

Mmu-let-7e sense target sequence:

5′-AAAC TA GCGGCCGC TAGT AACTATACAACCTCCTACCTCA T-3′

Mmu-let-7e antisense target sequence:

5′-CTAGA TGAGGTAGGAGGTTGTATAGTT ACTA GCGGCCGC TA GTTT-3

Mmu-let-7e scramble sense target sequence:

5′-AAAC TA GCGGCCGC TAGT AACTATACAACCTCCGGTATCA T-3′

Mmu-let-7e scramble antisense target sequence:

5′-CTAGA TGATACCGGAGGTTGTATAGTT ACTA GCGGCCGC TA GTTT-3′

Oligonucleotides pairs containing Pmel and Xba1 restriction sites (forward and reverse) of mouse FasL UTR region specific to mouse mmu-let-7e were generated by IDT DNA (IDT Inc). Both oligonucleotides (2 µl of each oligonucleotide) of normal or scramble mouse FasL UTR (specific to mmu-let-7e) regions were annealed in the presence of oligo annealing buffer (46 µl) at 90°C for 3 minutes and 37°C for 15 minutes. The annealed oligonucleotides of normal or scramble FasL UTR regions were used immediately for cloning into pmirGLO vector or stored at −20°C.

### Ligation and Transformation

Annealed oligonucleotides of normal or scramble FasL UTR were ligated to pmirGLO vector restricted with Pmel and Xba1 following the protocol of the company (Promega Corporation, Madison, WI). Ligated pmirGLO-FasL normal or pmirGLO-FasL- S UTR regions were transformed into competent bacterial (DH5 α) cells and positive clones were selected for further use after confirming the clones by sequencing. Positive selected clones were designated as pmirGLO-FasL for clones that contain normal FasL UTR and pmirGLO-FasL-S that contains scramble FasL UTR sequence.

### Transfection of EL4 Cells and Luciferase Assays

Freshly cultured EL4 cells (5×10^6^) were transfected with 5–10 µg of pmirGLO-FasL or pmirGLO-FasL-Scramble plasmids using Amaxa Nucleofector instrument and EL4 transfection kits from Lonza and following the protocol of the company (Lonza Cologne GMBH, Cologne, Germany). EL4 cells were also transfected independently with Pre-miR miRNA precursors of mmu-let-7e (MI0000561; PM12855) and anti-miR miRNA inhibitors (scramble mmu-let-7e) (MI0000561; AM12855) and negative controls for both from Applied Biosystems (Applied Biosystems) or in combination with pmirGLO-FasL or pmirGLO-FasL-S plasmids. We used Lipofectamine RNAMAX transfection kit and followed Reverse Transfection protocol of the company (Invitrogen). Two days post transfection, EL4 cells were replated in triplicate in 96-well plate (75 µl/well) and the cells were treated with vehicle or TCDD (100 nM/ml) and incubated for 24 h at 37°C, 5% CO_2_. Following treatments with vehicle or TCDD, luciferase assays were performed using Dual-Glo Luciferase Assay system from Promega and following the protocol of the company (Promega Corporation, Madison, WI). In brief, equal volume (75 µl/well) of Dual-Glo reagent was added to each well and thoroughly mixed. The cells were incubated for 10–15 minutes at room temperature to allow for cell lysis to occur. Firefly luciferase activity was measured by reading the sample luminescence using Victor^2^ (Perkin Elmer). After first reading of the samples, Dual-Glo Stop & Glo reagent (75 µl/well) was added to each well, mixed thoroughly, and incubated for 10–15 minutes. Renilla luminescence was measured by reading the sample luminescence using Victor^2^ (Perkin Elmer). Ratio of luminescence from experimental samples to luminescence from the control reporter was calculated. Luminescence ratio was then normalized to the ratio of control wells. Relative luminescence ratio was calculated from the normalized ratios and values were expressed as “normalized-fold induction.”

### Reverse Transcriptase PCR (RT-PCR) to Determine the Expression of AhR, CYP1A1, Fas, and FasL in Fetal Thymocytes

Total RNA from fetal thymocytes treated with TCDD or vehicle was isolated using RNeasy mini kit from Qiagen and following the protocol of the company (Qiagen, Valencia, CA). First strand cDNA synthesis was performed on total RNA (2 µg) and using iScript Kit and following the protocol of the company (Bio-Rad). To detect the expression of AhR, CYP1A1, Fas, and FasL, sets of primers specific to mouse AhR, CYP1A1, Fas, and FasL were used and PCR was performed as described earlier [38]. The PCR products, generated from mouse AhR, CYP1A1, Fas, and FasL primer pairs, were normalized against PCR products generated from mouse 18S forward (5'-GCCCGAGCCGCCTGGATAC-3') and reverse (5'-CCGGCGGGTCAT GGGAATAAC-3') primers after electrophoresis on 1.5% agarose gel and visualization with UV light. The band intensity of PCR products was determined using BioRad image analysis system (BioRad, Hercules, CA).

### Statistics

Statistical analyses were performed using GraphPad Prism software (San Diego, CA).

Differential (upregulated or downregulated) expression of miRs was analyzed using 2-sample t-test method. The significance of analysis of microarrays was performed using Kaplan-Meier method. Student’s t-test was also used for paired observations if data followed a normal distribution to compare TCDD-induced expression and quantification of CYP1A1 and other genes in thymocytes. Multiple comparisons were made using ANOVA (one-way analysis of variance) test and Tukey-Kramer Multiple Comparisons Test. P-value of ≤0.05 was considered to be statistically significant.
